# Spatial immunoprofiling of retroperitoneal leiomyosarcomas reveals intratumoral heterogeneity in immune cell infiltration, checkpoint molecule expression, and tertiary lymphoid structures

**DOI:** 10.1080/07853890.2025.2568725

**Published:** 2025-10-13

**Authors:** Iva Benesova, Jan Balko, Vira Tovazhnianska, Michal Rataj, Robert Lischke, Jirina Bartunkova, Katerina Kopeckova, Tomas Buchler, Winan van Houdt, Yvonne Schrage, David Moura, Javier Martin Broto, Zuzana Ozaniak Strizova, Andrej Ozaniak

**Affiliations:** ^a^ Department of Immunology, Second Faculty of Medicine, Charles University and University Hospital Motol, Prague, Czech Republic; ^b^ Department of Pathology and Molecular Medicine, Second Faculty of Medicine, Charles University and University Hospital Motol, Prague, Czech Republic; ^c^ Third Department of Surgery, First Faculty of Medicine, Charles University and University Hospital Motol, Prague, Czech Republic; ^d^ Department of Oncology, Second Faculty of Medicine, Charles University and University Hospital Motol, Prague, Czech Republic; ^e^ Department of Surgical Oncology, Netherlands Cancer Institute - Antoni van Leeuwenhoek Hospital (NKI-AVL), Amsterdam, the Netherlands; ^f^ Research Health Institute of Fundación Jiménez Díaz (IIS/FJD, UAM), Madrid, Spain; ^g^ Medical Oncology Department, Fundacion Jimenez Diaz University Hospital, Madrid, Spain; ^h^ Department of Oncology, University Hospital General de Villalba, Madrid, Spain

**Keywords:** Leiomyosarcoma, immune checkpoint inhibitors, tumour heterogeneity, lymphocyte activation gene 3, immune cell infiltration

## Abstract

**Introduction:**

Leiomyosarcoma (LMS) is a rare, aggressive cancer with limited treatment options at the metastatic stage. The response to immune checkpoint inhibitors (ICIs) is inconsistent, likely due to intratumoral heterogeneity, which is more pronounced in large tumours such as retroperitoneal LMS.

**Methods:**

This study examined heterogeneity in four large treatment-naive LMS tumours (ten samples per tumour) by analysing immune cells, tertiary lymphoid structures (TLSs), checkpoint molecules, and cytokine secretion across different tumour regions.

**Results:**

Significant region-dependent differences were observed in immune components, with TLSs present only at tumour margins and inconsistently across samples from the same tumour. Expression levels of programmed cell death 1 (PD-1) and programmed death ligand 1 (PD-L1) varied within individual tumours, and shared immune patterns were identified in specific regions, including elevated indoleamine 2,3-dioxygenase 1, absence of a particular macrophage subpopulation, and reduced PD-1 and lymphocyte activation gene 3 (LAG-3) expression at organ-adjacent margins. Anti-LAG-3 blockade altered cytokine and checkpoint molecule levels in a region-specific manner.

**Conclusion:**

These findings highlight substantial intratumoral heterogeneity, which may contribute to the variable response to ICI therapy. As immune checkpoint molecule expression influences treatment eligibility, multiple biopsies from different tumour regions may be necessary to assess immune infiltration accurately and guide therapy decisions.

## Graphical Abstract

**Figure:**
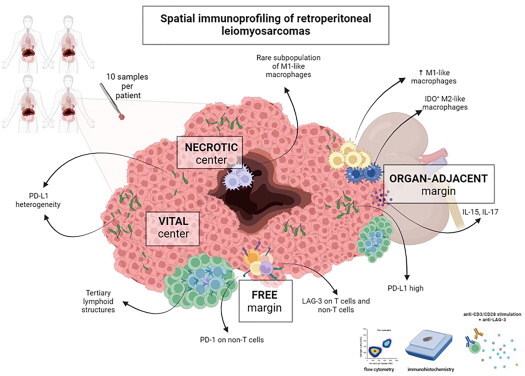

## Background

Leiomyosarcoma (LMS) accounts for 10%–20% of the soft-tissue sarcoma (STS) cases. A significant proportion of these tumours is found in the retroperitoneum or intra-abdominal regions [1–3]. The tumour grade, size, and primary localization are key determinants of overall survival [4]. These tumours are rare and require a multidisciplinary treatment approach. Surgery with macroscopically complete resection remains the main curative treatment option in localized disease [1]. Achieving complete surgical resection (R0) is crucial, as it directly influences long-term outcomes and recurrence rates [5]. The lung and liver are common sites of metastasis in retroperitoneal LMS, with a median survival time post-metastasis of approximately 20 months [1,6]. The role of radiotherapy or chemotherapy remains controversial, with significant differences in their use among sarcoma centres. The impact of radiotherapy on abdominal recurrence-free survival in patients with primary retroperitoneal sarcoma was evaluated in both the STRASS (Surgery plus Radiotherapy for Retroperitoneal Sarcoma Study) and STREXIT (Surgical Treatment of Retroperitoneal Sarcoma: Evaluation of Experimental Interventions and Therapies) trials [7], in which the STREXIT study demonstrated the clinical benefits of preoperative radiotherapy. However, the STRASS trial identified a positive effect of radiotherapy primarily in histological subtypes other than LMS [8]. Currently, cytotoxic chemotherapy yields suboptimal clinical outcomes, despite being the standard first-line approach for metastatic STS [9]. The potential benefit of neoadjuvant chemotherapy is currently under investigation in the STRASS II trial [10,11]. A recent study demonstrated that combination therapies involving doxorubicin and additional agents resulted in improved overall survival and progression-free survival compared to single-agent regimens [12]. These findings were further supported by evidence highlighting the good tolerability of combination therapies [9]. The LMS-04 study showed promising results, in which the combination of trabectedin and doxorubicin in first-line treatment doubled the median progression-free survival of patients with metastatic or unresectable LMS patients compared to doxorubicin alone [13]. Nevertheless, retroperitoneal LMS remains a significant clinical challenge, and the integration of immunotherapy into the treatment paradigm could provide substantial therapeutic benefits [1,14].

The advent of immune checkpoint inhibitors (ICIs) highlights the significance of the tumour microenvironment in cancer treatment. ICIs are a subtype of immunotherapeutics that potentiate anti-tumour immune response by blocking inhibitory pathways in immune cells, mainly T cells. Cytotoxic T lymphocyte-associated protein 4 (CTLA-4) together with PD-1 and PD-L1 can be blocked by antibodies such as ipilimumab (anti-CTLA-4), nivolumab (anti-PD-1), and pembrolizumab (anti-PD-1). This immune checkpoint blockade restores T-cell functions and facilitates immune-mediated tumour clearance. Emerging predictive biomarkers, including tumour mutational burden and tertiary lymphoid structures (TLSs), are increasingly recognized for their role in ICI response. ICIs have become a standard first-line therapy for various solid tumours, including early-stage diseases [15,16]. However, in STS, the response rate to immunotherapy remains low at approximately 15% [14]. Several combinations of ICI therapy (monotherapy, dual blockade, and combinations with tyrosine kinase inhibitors, chemotherapy, or radiation therapy) have shown unconvincing results. However, doublet-based chemotherapy combined with anti-PD1/PDL-1 inhibitors has demonstrated a significant objective response rate [17,18]. Concerning immunotherapy approaches, both as standalone treatments or in combination, in metastatic, PD-L1-positive, treatment-naive STS, including LMS, nivolumab plus ipilimumab demonstrated a higher response rate and longer progression-free survival compared to nivolumab alone. However, LMS is currently identified as a poorly sarcoma immunoresponsive subtype [19,20]. Given that most preclinical investigations and clinical trials evaluate multiple histological subtypes together, deciphering the specific determinants of LMS is crucial for selecting patients who would benefit from current or novel immunotherapy.

T cells are the most well-studied immune cell subset in STS and traditional targets of ICIs. The tumour microenvironment (TME) includes cytotoxic CD8^+^, helper, and regulatory CD4^+^ T cells, as well as subpopulations defined by activation or exhaustion states. Exhausted T cells express inhibitory molecules, such as programmed cell death 1 (PD-1), T-cell immunoglobulin and mucin domain-containing-3 (TIM-3), and lymphocyte activation gene 3 (LAG-3), and lose their effector functions. A stem cell-like subpopulation of progenitor exhausted T cells retains proliferation capacity, expresses fewer inhibitory markers, mediates antitumor immunity, and correlates with favourable prognosis [21].

Tumour-associated macrophages usually outnumber T cells in STS [22]. These cells exhibit high plasticity, with M1-like and M2-like phenotypes being the extremes of the phenotypic spectrum [23].

TLSs are ectopic lymphoid aggregates in non-lymphoid tissues that form during chronic inflammation, infection, or cancer. The densities, compositions, and maturations of these cells vary, influencing the antitumor immune response. TLS are generally predictors of immunotherapy response [16]. In other tumour types, the prediction of immunotherapy response currently relies on PD-L1 protein expression, measured through immunohistochemistry. PD-L1 expression is generally associated with an improved response rate to treatment with anti-PD-1 and anti-PD-L1 therapy; however, some patients, despite high expression of these molecules, do not respond [24]. Owing to the very low PD-L1 expression in nearly all sarcoma types, ICI therapy has not been incorporated into clinical practice for the treatment of STS [25].

Little is known about intratumoral heterogeneity in large LMSs. In this study, we aimed to characterize the tumour microenvironment by analysing multiple tumour regions. These regions included vital and necrotic centres, as well as organ-adjacent and free margins. To achieve this, we applied advanced methods to assess T cell and macrophage infiltration, checkpoint molecule expression, and TLS densities. The objective was to gain insights that are crucial for understanding the complex interplay within the TME and the diverse responses to ICIs within large tumours.

## Methods

### Patient samples

This prospective study included four treatment-naive patients with large conventional primary retroperitoneal LMS without any other malignancies or immunological disorders. Ten samples were collected from each patient. All patients underwent surgical resection at the Third Department of Surgery of Charles University and University Hospital Motol, Prague. All patients provided written informed consent, and the study was approved by the Ethics Committee of the Motol University Hospital (EK-563/22) and reporting was conducted in accordance with STROBE guidelines. Details regarding patient age, tumour stage, grade, tumour size, and patient outcomes are provided in Supplementary Table 1. All tumour samples were positive for SMA, H-caldesmon and desmin; focally weak positivity of EMA was also detected in all cases. The proliferation rate ranged from 15% to 40% as assessed by Ki-67. All samples were negative for CK AE1/3 and S100. Tumour samples were evaluated by a sarcoma-experienced pathologist, who defined four regions: vital centre (viable central part of the tumour without necrotic foci), necrotic centre (central part of the tumour showing necrotic changes but containing viable tumour cells), free margin (peripheral part of the tumour without any contact to visceral organs), and organ-adjacent margin (peripheral part in proximity or infiltrating a visceral organ, e.g. kidney or large intestine) **(**Figure S1**).** Moreover, the tumour grade was separately evaluated for each sample to assess the variability between tumour samples within a single tumour tissue (Table S2).

### Immunohistochemistry

Formalin-fixed, paraffin-embedded tumour samples were stained for PD-1 (*n* = 36) and PD-L1 (*n* = 35) expression. Tumour regions unsuitable for analysis due to complete necrosis were excluded. TLSs were identified in the tumour regions. The tumour samples were fixed in neutral-buffered 4% formaldehyde and postfixed. The paraffin blocks were sectioned into 3-µm-thick histologic sections and stained using the following antibodies and protocols: anti-PD1 antibody (clone: polyclonal serum, Bio-Rad, Hercules, CA, USA, dilution 1:200, without antigen retrieval), and anti-PD-L1 antibody (mouse monoclonal antibody, clone: 22C3, Agilent Dako, certified kit, processed according to the certified Autostainer Link 48 protocol). Detection of PD-1 was performed using a one-step micropolymeric non-biotin system (Bio SB, Santa Barbara, CA, USA) with a peroxidase and 3,3′-diaminobenzidine tetrahydrochloride solution. Nuclei were counterstained with haematoxylin. The slides were manually evaluated and 30 high-power fields (HPFs) were counted starting from hot spots. The counted HPFs were converted into cell densities. Additionally, PD-L1 staining was classified throughout the entire sample. Complete or partial membrane positivity, regardless of intensity was considered positive. Based on the percentage of positive tumour cells, each sample was semi-quantitatively classified into three categories: no expression (< 1% of positive cells), intermediate expression (1% to 49% of positive cells), and high expression (> 50% of positive cells). After evaluating the entire sample, TLS were identified as lymphoid follicles, with and without germinal centres on haematoxylin and eosin-stained samples.

### Cell isolation from tumour tissue

Tumour tissues were processed into single-cell suspensions by mechanical dissection and enzymatic dissociation for flow cytometry and functional analyses. Tumour tissues were transferred into gentleMACS^TM^ C tubes (Miltenyi Biotec, Bergisch Gladbach, Germany) and cut into small pieces using scissors. Subsequently, RPMI 1640 medium (Thermo Fisher Scientific, Waltham, MA, USA) supplemented with 1 mg/mL collagenase I (Roche, Basel, Switzerland) and 0.05 mg/mL DNase I (Roche) was added for further dissociation using the gentleMACS^TM^ dissociator (Miltenyi Biotec) at program h_tumour_01. The suspension was digested for 30 min at 37 °C followed by dissociation in gentleMACS^TM^ at program h_tumour_01 and passed through a 100-µm nylon cell strainer (Corning, Inc., Corning, NY, USA). The cell suspension was washed in phosphate-buffered saline (PBS), and red blood cells were lysed for 10 min at room temperature (RT), before centrifuging at 300 × *g* for 5 min at RT. The cells were cryopreserved until further use.

### Flow cytometry

The phenotypes of T cells (*n* = 37) and macrophages (*n* = 26) were evaluated using flow cytometry. Isolated cells were stained using the Zombie NIR^TM^ Fixable Viability Kit (Biolegend, San Diego, CA, USA, 1:750) for 20 min at RT in the dark. Then, the cells were washed in PBS, centrifuged, and incubated in a mixture of fluorescently labelled monoclonal antibodies (Table S3) in PBS for 20 min at 4 °C in the dark. The cells were washed in PBS and the T cell panel was analysed using the BD LSRFortessa cytometer. The cells in the macrophage panel underwent intracellular staining using the eBioscience^TM^ Intracellular Fixation & Permeabilization Buffer Kit (Thermo Fisher Scientific), following the manufacturer’s recommendations. IC fixation buffer was added and the cell suspension was vortexed, followed by 30 min of incubation at RT in the dark. Next, the sample was washed twice in 1× permeabilization buffer and centrifuged at 400 × *g* for 5 min at RT. The antibody mixture (Table S3) in 1× permeabilization buffer was incubated for 30 min at RT in the dark, followed by washing in 1× permeabilization buffer and centrifugation at 400 × *g* for 5 min at RT. The cells were resuspended in PBS and measured using the BD LSRFortessa cytometer. Subsequent analyses were performed using FlowJo (BD Bioscience, v10.09). Fluorescence minus one and isotype controls were used where necessary to set proper gates. Additionally, dimensionality reduction and clustering analyses were performed. CD4^+^ T cells, CD8^+^ T cells, and macrophages were gated separately, downsampled (v3.3.1), and concatenated. Uniform Manifold Approximation and Projection (UMAP, v4.0.3) [26], FlowSOM (v3.0.18) [27], and cluster explorer (v1.7.6) plugins in FlowJo were used.

### Selective T-cell stimulation and anti-LAG-3 blockade

T cells isolated from the tumour were stimulated with anti-CD3/CD28 Dynabeads^TM^ (Thermo Fisher Scientific, Waltham, MA, USA) in the presence or absence of anti-LAG-3 monoclonal antibodies (Selleck Chemicals, Houston, TX, USA). Cytokine secretion was analysed in the supernatants after 24 h of culture. The response to stimulation with/without anti-LAG-3 blockade (Selleck Chemicals, Houston, TX, USA) was evaluated in different tumour regions. The tumour-isolated cells were divided into three groups; unstimulated, anti-CD3/CD28 stimulated with Dynabeads^TM^ Human T-Activator CD3/CD28 (Thermo Fisher Scientific, 10 µL/mL), and anti-CD3/CD28 Dynabeads^TM^ stimulated + anti-LAG-3 (10 µg/mL). Some samples were divided into two groups or were not used owing to a low total cell count. Then, 2 × 10^5^ tumour-isolated cells were cultivated for 24 h at 37 °C, 5% CO_2_ in 200 µL of complete medium (RPMI 1640 supplemented with 10% heat-inactivated foetal bovine serum (Cytiva Sweden AB, Uppsala, Sweden), 100 U/mL penicillin-streptomycin, and 2 mM GlutaMax (Thermo Fisher Scientific). The samples were centrifuged (300 × *g*, 5 min, RT) and the supernatant was collected for the multiplex Luminex cytokine bead-based assay.

#### Multiplex luminex cytokine bead-based assay

The levels of cytokines in the supernatants were measured using the Human XL Cytokine 16-Plex Kit (Bio-techne, Minneapolis, MN, USA). The panel included C-C motif chemokine ligand (CCL)2, CCL5, C-X-C motif chemokine ligand 10 (CXCL10), Granzyme B, interferon gamma (IFN-γ), interleukin (IL)-2, IL-8, IL-10, IL-15, IL-17, macrophage inflammatory protein (MIP)-1α, MIP-1β, PD-L1, tumour necrosis factor alpha (TNF-α), TNF-related apoptosis inducing ligand (TRAIL), and vascular endothelial growth factor (VEGF). The custom Human XL Cytokine 16-Plex Kit was utilized according to manufacturer’ protocol. Briefly, 25 µL of the supernatant was diluted 1:1 in the calibration diluent. A microparticle cocktail was added and the whole suspension was incubated on a shaker at 800 rpm for 24 h at 4 °C. The samples were washed three times and biotin-antibody cocktail was added for incubation for 1 h at RT on a shaker at 800 rpm. After washing, streptavidin-PE was added for 30 min at RT on the shaker at 800 rpm. The samples were washed again and analysed using the Luminex 200 system and Bellisa software (Merck Millipore, Burlington, MA, USA).

### Data analysis

Differences in frequencies of specific immune cell subsets between tumour tissues of different patients (defined as interpatient variability)/tumour regions within a single tumour tissue (defined as intratumoral heterogeneity) were tested after evaluating the distribution of data using the Shapiro–Wilk normality test. Ordinary one-way ANOVA and Tukey’s multiple comparison tests were utilized for parametric data and Kruskal–Wallis tests with Dunn′s multiple comparison tests were used to evaluate non-parametric data. To ensure that the data from different patients were comparable, in patient D, three samples from the vital centre of the tumour were taken, averaged, and this average was used for the T cell analysis. For the comparisons of mean fluorescence intensity (MFI), pg/mL, and fold changes of cytokines between unstimulated and stimulated supernatants or stimulated and stimulated + anti-LAG-3 supernatants, multiple Wilcoxon tests with the false discovery rate (FDR) method to correct for multiple comparisons were performed. For comparisons of cytokine secretion under various conditions (unstimulated/stimulated/stimulated + anti-LAG-3 samples) at different tumour regions, two-way mixed-effects model with the Geisser-Greenhouse correction and Tukey’s multiple comparison tests were utilized. The statistical analyses and data visualization were performed using GraphPad Prism 10.0.3 (GraphPad, Boston, MA, USA) and *p* ≤ 0.05 was considered significant. All column graphs are presented with means and standard deviations. Factor analysis of mixed data (FAMD) was primarily used to examine clustering patterns of individual samples to determine whether samples are related within the same patient and tumour region. Data were analysed using RStudio and R (v4.3.2) with the packages FactoMineR [28],factoextra [29], ggplot2 [30], and ggrepel [31].

## Results

### Characterization of immune cell subsets in LMS

**Figure 1.**
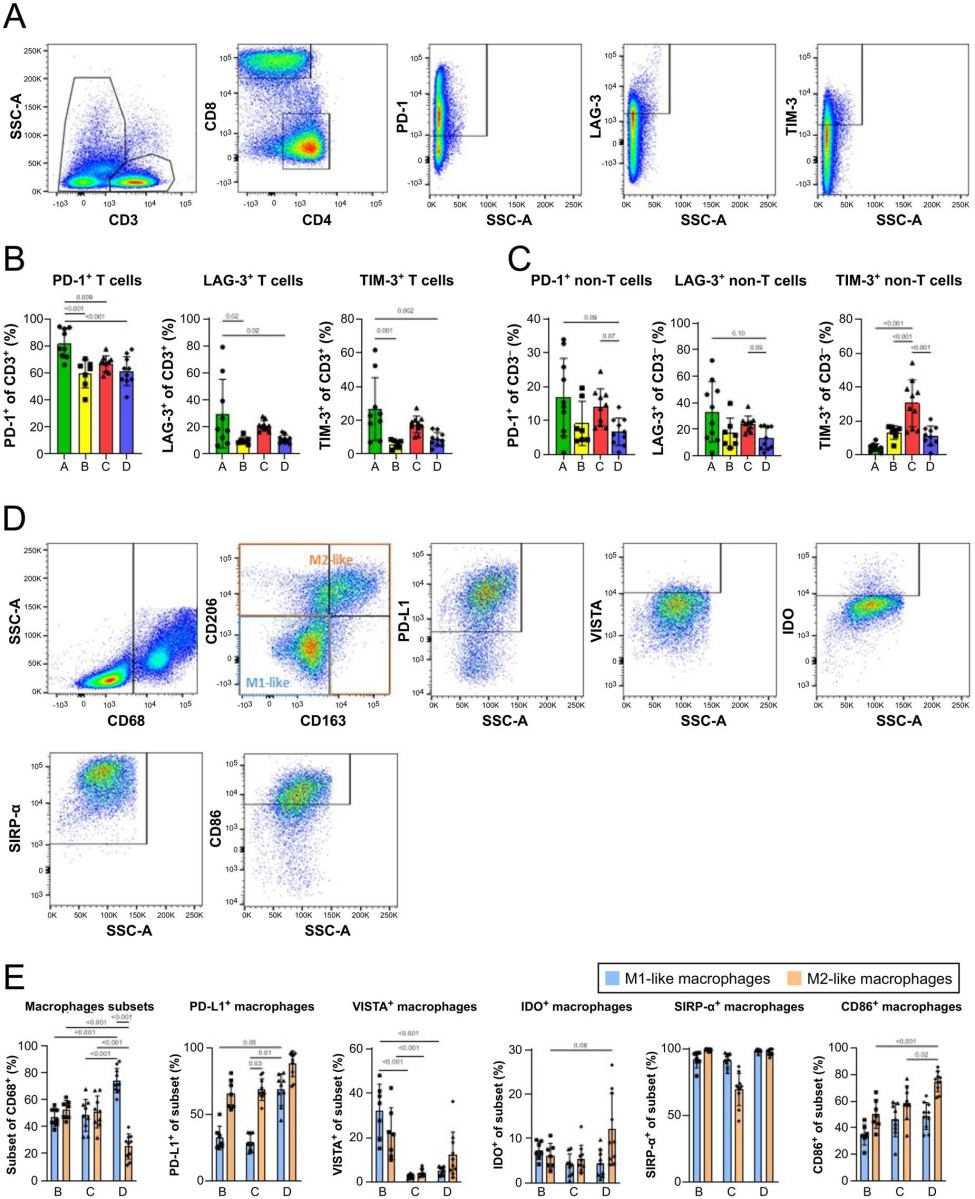
Characterization of immune cell infiltration in patients with leiomyosarcomas (LMS). (A) gating strategy for the T cell analysis using flow cytometry. Representative gates for PD-1, LAG-3, and TIM-3 are set for CD3^+^ T cells. Characterization of immune checkpoint molecule expression on (B) CD3^+^ T cells and (C) CD3^-^ non-T cells (*n* = 36). (D) Gating strategy for the macrophage analysis using flow cytometry. Representative gates for macrophage markers were set for M2-like macrophages (CD163^-^CD206^+^, CD163^+^CD206^+^, CD163^-^CD206^+^, orange gate). (E) Characterization of various macrophage markers on M1-like (CD68^+^CD163^-^CD206^-^) and M2-like macrophages in patients with LMS (*n* = 26). Significance for macrophage-related molecules is depicted between M1-like subsets and between M2-like subsets in various patients. The difference in frequencies was analysed by one-way ANOVA with Tukey’s multiple comparisons tests for parametric data or by Kruskal–7uallis test with Dunn’s multiple comparisons test for non-parametric data. *p* ≤ 0.05 was considered significant.

T cells are classic targets of ICIs. Therefore, we evaluated the expression of PD-1, LAG-3, and TIM-3 in T cell subsets (Figure 1(A)). Interpatient variation was observed (Figure 1(B)). PD-1 expression on CD3^+^ T cells (mean: 59.4%–81.9%) displayed higher rates of positivity than other analysed inhibitory molecules; LAG-3 (mean: 9.9%–29.7%) and TIM-3 (mean: 5.9%–26.7%), which were expressed at much lower frequencies. This pattern was consistent across all patients. Accumulating evidence emphasizes the presence of immune checkpoint molecules in non-T cell subsets, where they appear to play important, though often less well-understood, roles [32]. Therefore, we also analysed the expression of immune inhibitory molecules in these cells as well (Figure 1(C)). The rates of PD-1 were lower than those for T cells, but they were still noteworthy (mean: 6.8%–17%). The expression of LAG-3 (mean: 13%–32%) was similar in non-T cell and T cell subsets, and this pattern was consistent across patients. The expression of TIM-3 (mean: 4.3%–30.5%) varied between T cells and non-T cells in each patient.

We further classified macrophages into M1-like (CD68^+^CD163^-^CD206^-^) and M2-like (CD68^+^CD163^-^CD206^+^/CD163^+^CD206^+^/CD163^+^CD206-) subtypes and evaluated the expression of macrophage-associated molecules (Figure 1(D)). Although one would expect a predominance of pro-tumorigenic M2 macrophages, no significant shift towards the M2-like phenotype was observed across the study cohort (Figure 1(E)). Moreover, patient D had significantly higher M1-like macrophage counts. PD-L1 expression was predominantly associated with M2-like macrophages. However, a subset of M1-like macrophages also displayed PD-L1 positivity. The other analysed molecules were expressed at varying levels in both macrophage subsets.

**Figure 2.**
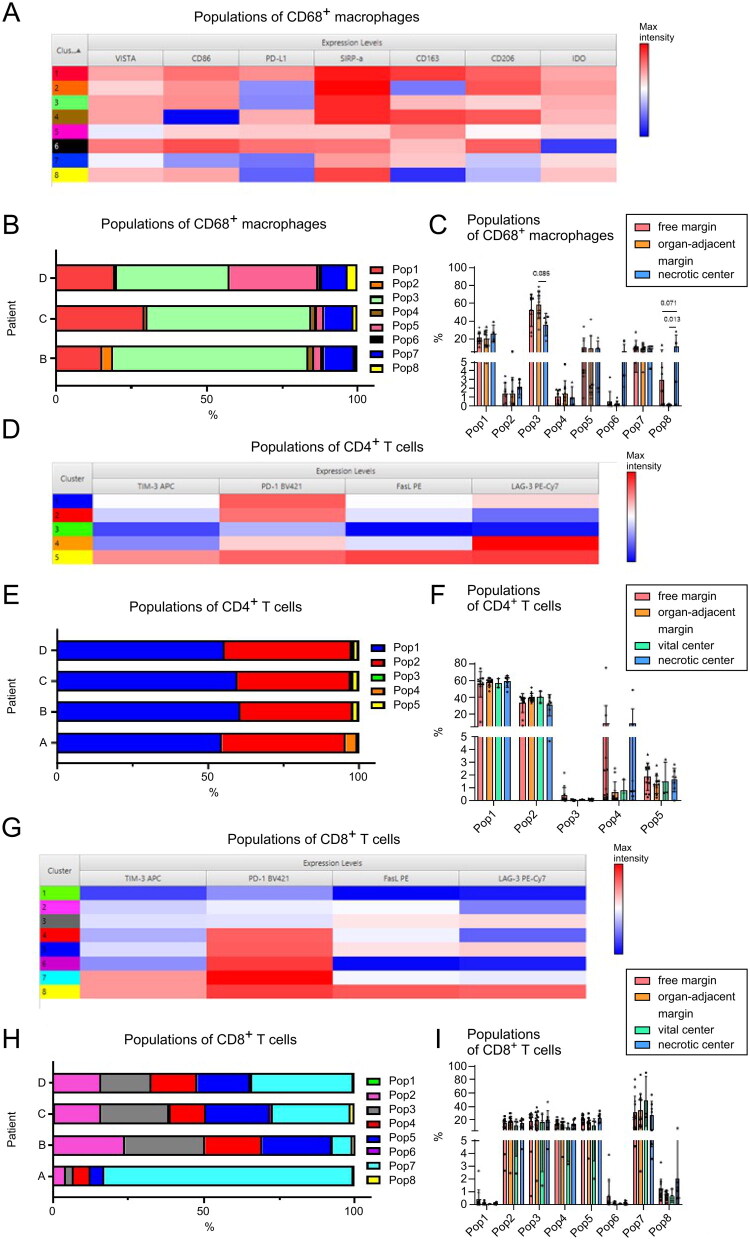
Characterization of T cell and macrophage subpopulations in patients with LMS. (A) FlowSOM and cluster explorer analyses revealed eight macrophage subpopulations. The heatmap depicts the intensity of each marker (red indicates high, blue indicates low) within each subpopulation (1–8, left side). (B) Distribution of macrophage subpopulations in individual patients (*n* = 21). (C) Distribution of macrophage subpopulations at different tumour regions. (D) FlowSOM and cluster explorer analyses revealed five subpopulations of CD4^+^ T cells. (E) Distribution of CD4^+^ T cell subpopulations in individual patients (*n* = 34). (F) Distribution of CD4^+^ T cell subpopulations at different tumour regions. (G) FlowSOM and cluster explorer analyses revealed eight subpopulations of CD8^+^ T cells. Subpopulations that share similar pattern expression with CD4^+^ T cell subpopulations are marked by the same colour. (H) Distribution of CD8^+^ T cell subpopulations in individual patients (*n* = 34). (I) Distribution of CD8^+^ T cell subpopulations at different tumour regions. In C, F, I, the samples from each patient are represented by a special symbol: patient A, circle; patient B, square; patient C, triangle; patient D, rhombus. To maintain the sample balance, average values for three samples from the vital centre of patient D are presented in the T cell analysis. The difference in frequencies was analysed by one-way ANOVA with Tukey’s multiple comparisons test for parametric data or by Kruskal–Wallis test with Dunn’s multiple comparisons test for non-parametric data. *p* ≤ 0.05 was considered significant.

These findings suggest phenotypic plasticity of macrophages within the TME. To explore the subpopulations further, we applied unsupervised techniques to analyse marker co-expression and expression intensities (Figure 2).

### Detailed identification of T-cell and macrophage phenotypes and subsets

The diverse phenotypes of macrophage subpopulations were defined using FlowSOM and cluster explorer analyses (Figure 2(A)). Among the eight main populations identified, two macrophage subsets—population 1 (red), characterized by high expression of SIRP-alpha, CD163, and CD206, and population 3 (green), characterized by low expression of PD-L1—comprised more than half of the macrophages in the patients included in the analysis (Figure 2(B)). Of note, population 8 (yellow) with low expression of PD-L1, CD163, and CD206, exhibiting an M1-like phenotype, was more abundant in the necrotic centre. These M1-like macrophages were, however, reduced in the free margin and almost undetectable in the organ-adjacent margin (*p* = 0.071 and *p* = 0.013, respectively) (Figure 2(C)). Four subpopulations of CD4^+^ T cells are depicted in Figure 2(D). The dominant subpopulations in all patients with LMS were populations 1 (blue) and 2 (red) (Figure 2(E)), characterized by high PD-1 expression and different levels of LAG-3 expression. Interestingly, triple positivity for PD-1, LAG-3, and TIM-3 (yellow population) was detected at very low levels (0.62%–1.96%). We did not observe significant differences in the subpopulation frequencies at distinct tumour regions (vital and necrotic centres and free and organ-adjacent margins) (Figure 2(F)).

In our analysis, we identified eight subpopulations of CD8^+^ T cells (Figure 2(G)). In these CD8^+^ subsets, PD-1 was highly expressed in all patients (Figure 2(H)). Moreover, a substantial proportion of cells co-expressed TIM-3 with PD-1. However, population eight (yellow), characterized by high expression of the three inhibitory molecules PD-1, TIM-3, and LAG-3, was relatively discrete in most patients. Of note, there was a relative increase in the prevalence of these CD8+ PD-1^+^TIM-3^+^LAG-3^+^ T cells within the necrotic tumour centre (Figure 2(I)). Importantly, three distinct CD8^+^ T cell populations exhibited high levels of LAG-3. One of these CD8^+^ LAG-3^+^ T cell populations (population 3, gray), defined by low TIM-3 and PD-1 expression, showed relatively high abundance in 75% of patients (16.6%–26.4% of CD8^+^ T cells in these three patients) (Figure 2(G,H)).

Similar to the results for CD4^+^ T cells, there were no significant differences in subpopulations among tumour regions (Figure 2(I)). Based on these observations, combinatorial immunotherapies targeting immune inhibitory molecules (PD-1, TIM-3 and/or LAG-3) could be effective against LMS.

### Spatial distribution of PD-1, PD-L1 and TLS in LMS

**Figure 3.**
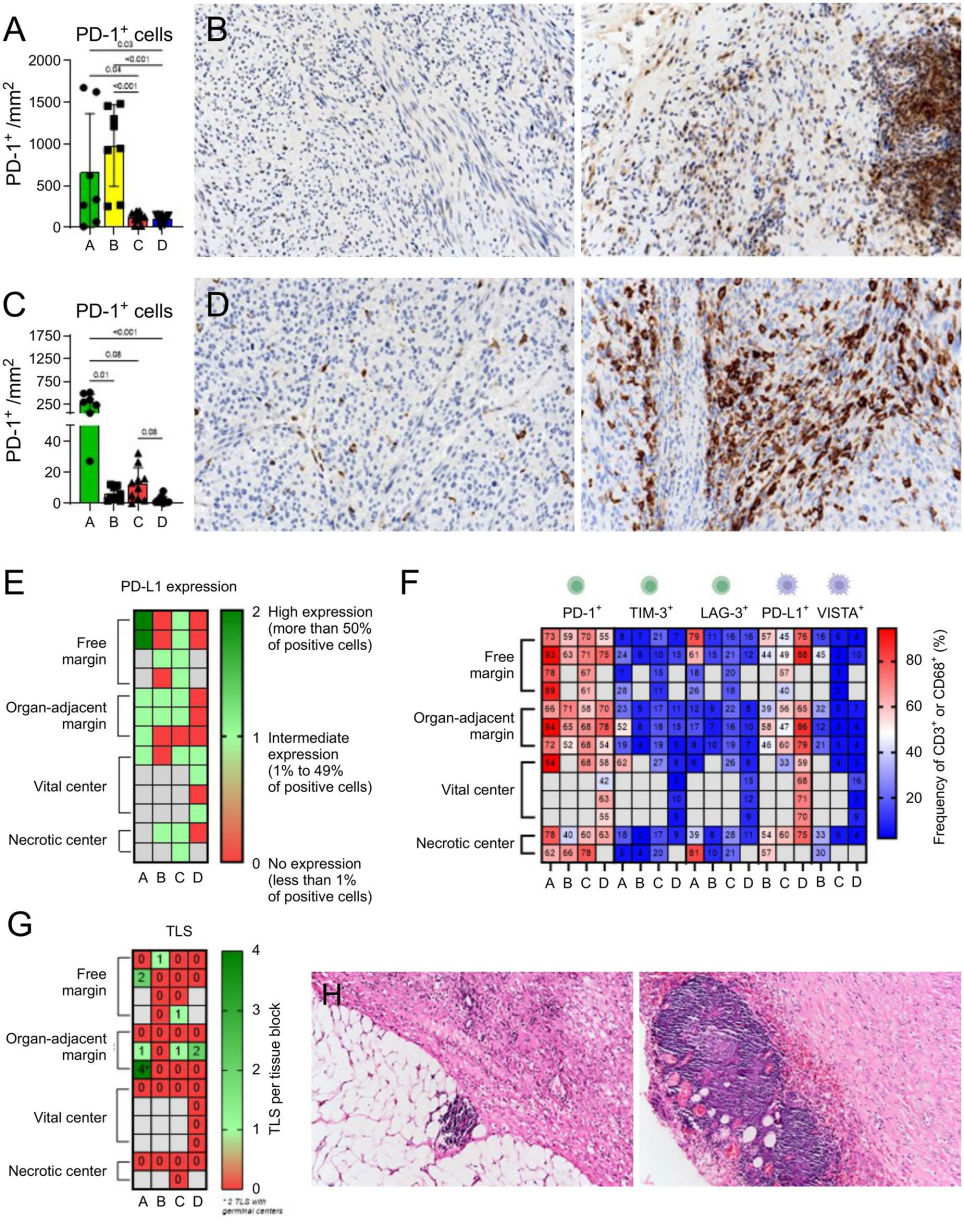
Intratumoral heterogeneity of LMS might influence the patient eligibility to certain immunotherapeutic approaches. (A) Graphical representation of difference in PD-1 within each patient and among all patients (*n* = 35); 30 high-power fields were counted for each tumour sample. (B) Immunohistochemical staining of PD-1 in leiomyosarcoma (LMS) tissue at different tumour regions in one patient. (C) Graphical representation of difference in PD-L1 within each patient and among all patients (*n* = 35). (D) Immunohistochemical staining of PD-L1 in LMS tissue at different tumour regions within one patient. (E) Semi-quantitative classification of samples based on PD-L1 positive tumour cells (*n =* 35). (F) Heatmap of flow cytometry data displays the differences in marker expression within all samples (*n* = 37). (G) Schematic illustration of tertiary lymphoid structures in each tumour sample (TLS) (*n* = 36). (H) Example of tertiary lymphoid structure with and without a germinal centre. *p ≤ 0.05, ***p ≤ 0.001.

Since IHC is used to evaluate immune checkpoint molecule expression and to determine patient eligibility for immunotherapy, we have used IHC to examine the spatial distribution of PD-1, PD-L1, and TLS. Surprisingly, each patient showed substantial heterogeneity in the expression of these molecules and in TLS prevalence. The most profound difference was observed in patient A, in whom PD-1 expression ranged from 9.28 to 1674.4/mm^2^ (180.4-fold higher expression) and PD-L1 ranged from 27.12 to 503.66/mm^2^ (18.6-fold higher expression) (Figure 3(A–D), Table S4). Because patient eligibility for immunotherapy is determined by the percentage of PD-L1-positive tumour cells, we systematically collected this information across different tumour regions (Figure 3(E)). Interestingly, we observed substantial intratumoral heterogeneity in PD-L1 expression (Figure S2). For instance, patient A exhibited high PD-L1 expression (> 50%) in two samples derived from the free margin. Other regions exhibited intermediate (PD-L1 expression. Patient B exhibited considerable heterogeneity in PD-L1 expression across and within tumour regions. In the free margin, three of four samples had no detectable PD-L1 expression, whereas one sample exhibited intermediate expression (1%–49%). Similar variability was observed in the other regions. Patient C predominantly exhibited intermediate PD-L1 expression across most tumour samples; however, one sample from the organ-adjacent margin displayed zero PD-L1 expression. Patient D showed no PD-L1 expression in the free margin, organ-adjacent margin and necrotic centre but demonstrated intermediate expression in the vital centre. The differences between other targetable molecules (PD-1, TIM-3, and LAG-3 on CD3^+^ T cells, PD-L1 and VISTA on CD68^+^ macrophages) that were analysed by flow cytometry are depicted in a heatmap (Figure 3(F)). Interestingly, in two patients, at least one tumour region exhibited low PD-1 expression, while other regions demonstrated high PD-1 expression within the same tumour. In contrast, in patient A, LAG-3^+^CD8^+^ T cells were highly positive in 3 of 10 regions (Figure 3(F)).

TLS are predictors of response to immunotherapy in many cancers [16]. Patient A exhibited at least one TLS per region in three of seven samples. Moreover, two out of four TLSs in the sample from the organ-adjacent margin had germinal centres. Patient C exhibited TLS in two of the ten samples, while patients B and D had only one specimen positive for the presence of TLS (Figure 3(G,H)). Interestingly, spatial differences in TLS did not correlate with tumour grade, as most samples exhibited consistent tumour grade (Table S2).

Given the remarkable diversity within a tumour, these findings further emphasize the need to analyse multiple samples from different regions in large tumours to select patients that could benefit from immunotherapy. Moreover, the use of multiple immunological targets can improve treatment outcomes in LMS.

### Analysis of region-specific immune cell populations within LMS

**Figure 4.**
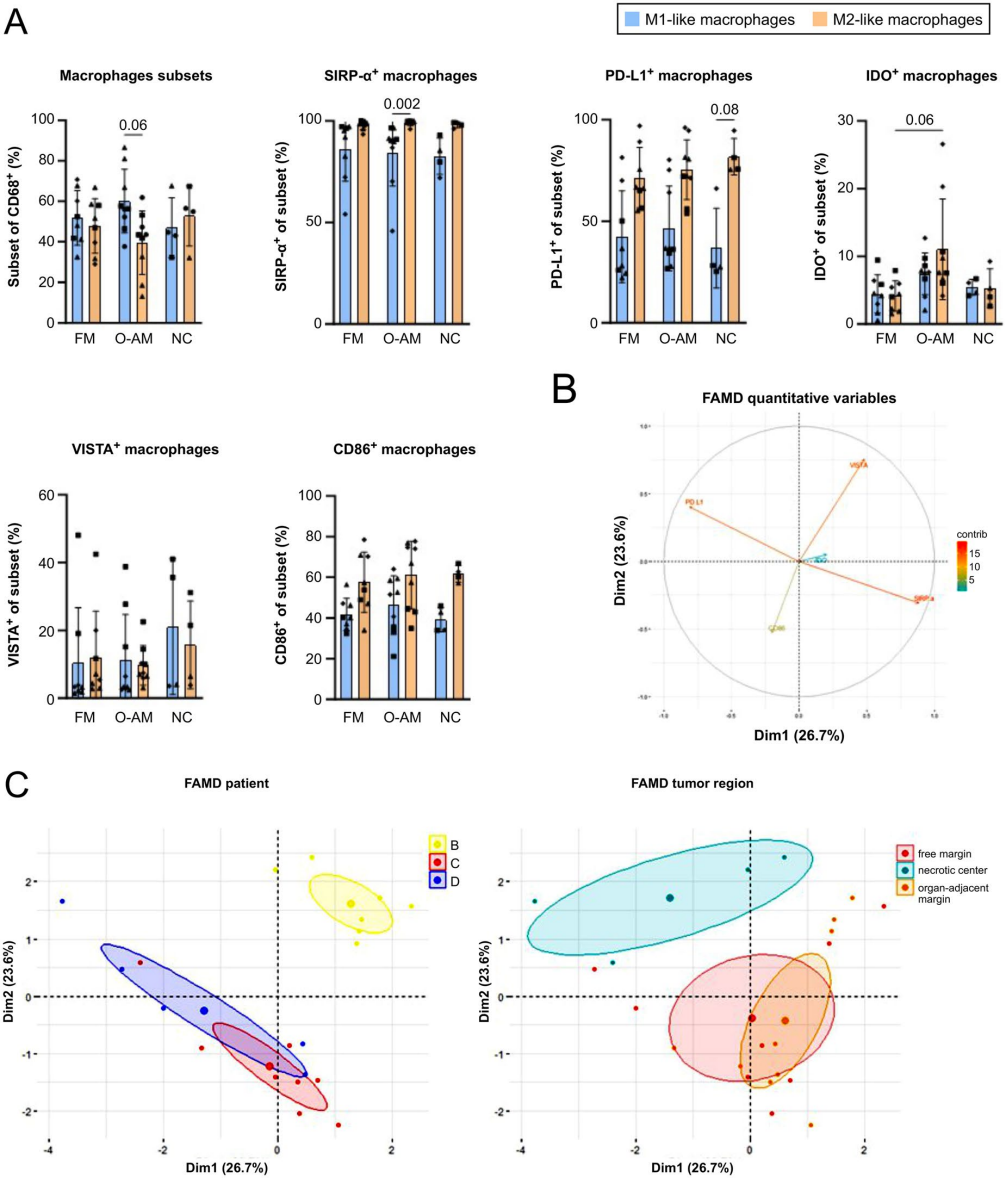
Region-specific immune profiles with a focus on macrophages. (A) Analysis of multiple molecules on M1-like (CD68^+^CD163^-^CD206^-^) and M2-like (CD163^-^CD206^+^, CD163^+^CD206^+^, CD163^-^CD206^+^) macrophages within three tumour regions (FM = free margin, O-AM = organ-adjacent margin, NC = necrotic centre). The samples from each patient are represented by a special symbol: patient B, square; patient C, triangle; patient D, rhombus. The difference in frequencies for each marker was analysed by one-way ANOVA with Tukey’s multiple comparisons test for parametric data or by Kruskal–Wallis test with Dunn’s multiple comparisons test for non-parametric data. *p* ≤ 0.05 was considered significant. Factorial analysis of mixed data (FAMD) for categorical (patient and tumour region) and continuous variables (CD86, IDO, PD-L1, SIRP-α, and VISTA). (B) Correlative circle marks quantitative variables; each arrow depicts the contribution of the variable to the first and second dimensions. The circle illustrates the correlations between variables (e.g. PD-L1 and SIRP-α are negatively correlated). (C) Individual points are plotted and coloured based on patient (B, yellow; C, red; D, blue) and location (free margin, red; necrotic centre, blue; organ-adjacent margin, orange). The largest point shows the average location of each group.

**Figure 5.**
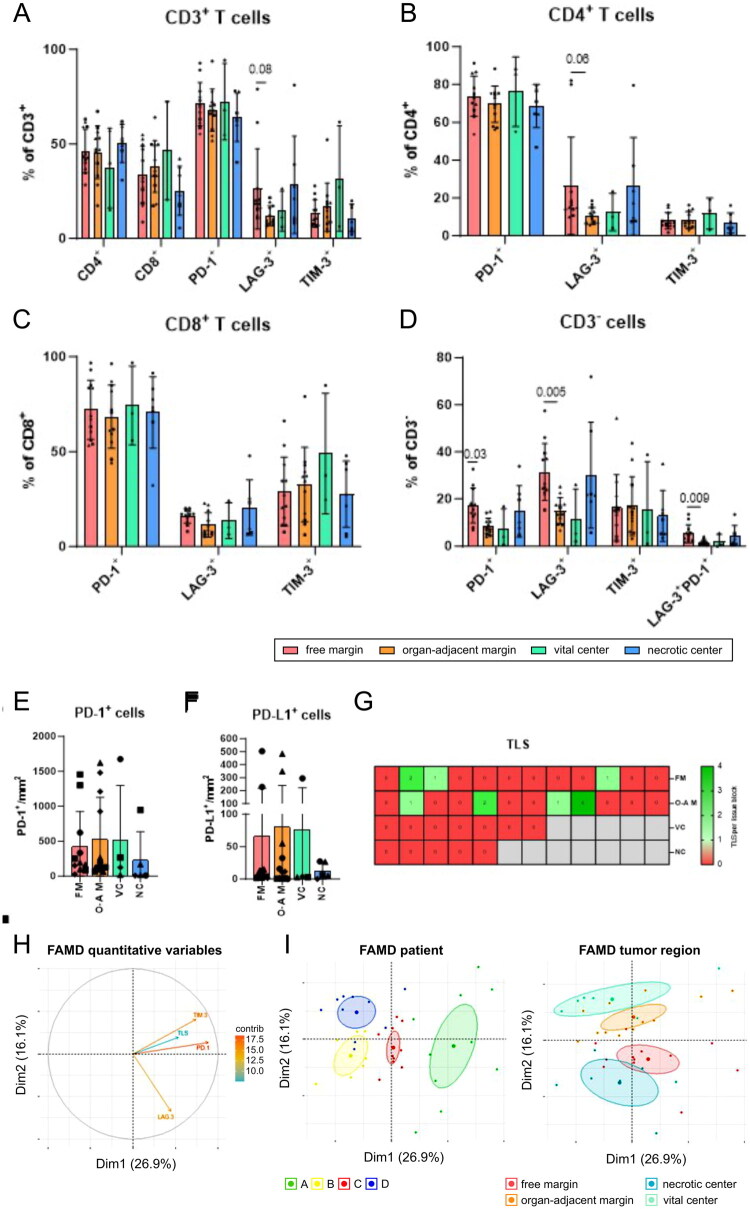
Dissection of the region-specific immune response with a focus on checkpoint molecules and TLS. Analysis of region-specific immune infiltrates on (A) CD3^+^ T cells, (B) CD4^+^ T cells, (C) CD8^+^ T cells, and (D) CD3^-^ cells by flow cytometry. (E) PD-1 and (F) PD-L1 expression at different tumour regions analysed by immunohistochemistry. (G) TLS presence divided by tumour regions. Samples from each patient are represented by a special symbol: patient A, circle; patient B, square; patient C, triangle; patient D, rhombus. To maintain the sample balance, average values of three samples from the vital centre of patient D are presented. The difference in frequencies for each marker separately was analysed by one-way ANOVA with Tukey’s multiple comparisons test for parametric data or by Kruskal–Wallis test with Dunn’s multiple comparisons test for non-parametric data. *p* ≤ 0.05 was considered significant. Factorial analysis of mixed data (FAMD) for categorical (patient and location) and continuous variables (PD-1, LAG-3, TIM-3, and TLS). (H) Correlative circle marks quantitative variables; each arrow depicts the contribution of the variable to the first and second dimensions. Moreover, the circle illustrates the correlations between the variables (e.g. PD-1 and TIM-3 are positively correlated). (I) Individual points are plotted and coloured based on patient (A, green; B, yellow; C, red; D, blue) and location (free margin, red; necrotic centre, blue; organ-adjacent margin, orange; vital centre, green). The largest point shows the average location of each group.

The tumour architecture is heterogeneous, and many factors, such as hypoxia, abnormal vascularity, genetic instability, and epigenetic modifications of tumour cells shape the immunity [33]. We observed differences in macrophage populations among tumour regions (Figure 2(C),); therefore, the tumour regions were investigated in more detail to decipher location-specific immune-related features (Figures 4, 5, Table S5). M1-like macrophages outnumbered M2-like macrophages in the organ-adjacent margin (*p* = 0.06) (Figure 4(A)). Higher levels of indoleamine-2,3-dioxygenase 1 (IDO) positive M2-like macrophages were detected in the organ-adjacent margin than in the free margin (*p* = 0.06). To determine whether the observed variations could be attributed to the patient or tumour region, a FAMD was conducted. We detected a negative correlation between VISTA and CD86 and between PD-L1 and SIRP-α (Figure 4(B)). Samples from patient B were separated from samples from the other patients, indicating that the tumour samples from this patient were different from those from patients C and D (Figure 4(C)). Patients C and D shared some overlap. Samples from the necrotic centre were clearly separated, indicating distinct macrophage populations in this tumour region.

Next, we compared immune checkpoint molecules among the tumour regions (Figure 5). LAG-3 levels were higher in CD3^+^ T cells (*p* = 0.08) (Figure 5(A)) and CD4^+^ T cells (*p* = 0.06) (Figure 5(B)) in the free margin compared to those in the organ-adjacent margin, whereas no difference was detected for CD8^+^ T cells (Figure 5(C)). Substantial differences were detected in CD3^-^ cells. PD-1 (*p* = 0.03) and LAG-3 (*p* = 0.005) positive cells were significantly more abundant in the free margin compared to the organ-adjacent margin (Figure 5(D)). The overall expression of PD-1 (Figure 5(E)) and PD-L1 (Figure 5(F)) assessed by the immunohistochemistry, regardless of cell type, did not exhibit significant differences. TLS was detected only at the tumour margins (Figure 5(G)). Lastly, we analysed the factors driving the variation among the samples using FAMD. The levels of PD-1, TIM-3, and TLS were positively correlated (Figure 5(H)). Patient-driven variability explained most the variation because the samples were well separated on individual plots. Additionally, tumour regions were also separated (Figure 5(I)), with an overlap between the vital centre and organ-adjacent margin. Moreover, samples from the free margin and necrotic centre shared similarities. These data indicate the presence of differences among tumour regions, suggesting that it may be beneficial to analyse various tumour regions to better understand immune infiltration within large LMSs.

### Differential cytokine responses to T-cell stimulation in tumour regions and patients

**Figure 6.**
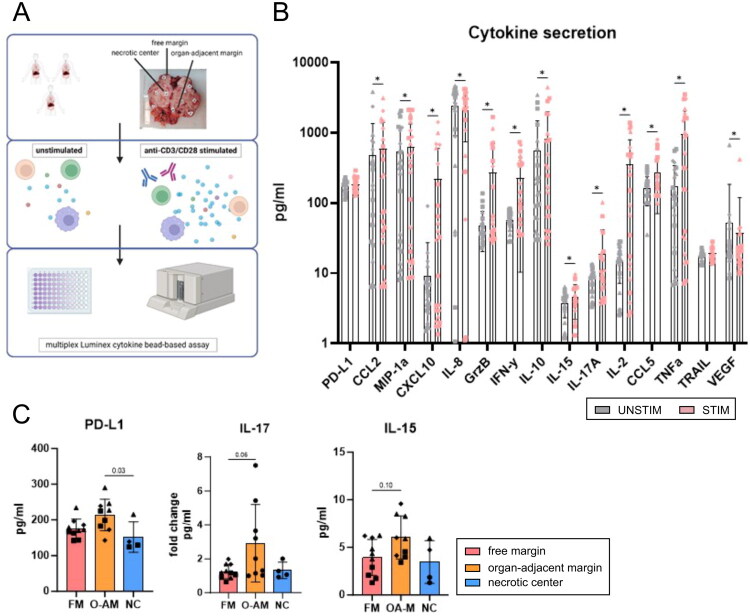
Reaction to anti-CD3/CD28 stimulation. (A) Experimental workflow. (B) Changes in cytokines after anti-CD3/CD28 stimulation for 24 h (*n* = 24). Comparisons were performed using multiple paired Wilcoxon tests and false discovery rates (FDR). FDR does not represent the actual *P*-value. (C) Tumour region-specific differences in cytokine expression (*n* = 23). Samples from each patient are represented by a special symbol: patient B, square; patient C, triangle; patient D, rhombus. The difference in frequencies for each marker was analysed by one-way ANOVA with Tukey’s multiple comparisons test for parametric data or by Kruskal–Wallis test with Dunn’s multiple comparisons test for non-parametric data. *p* ≤ 0.05 was considered significant. Image created using biorender.

Next, we evaluated differences in the reactions of tumour regions to stimulation (Figure 6). Moreover, we investigated the intratumoral heterogeneity (interpatient variability) in response to stimulation. Accordingly, 24 h of anti-CD3/CD28 stimulation was performed on the tumour regions (free margin, organ-adjacent margin, and necrotic centre) of three patients (Figure 6(A)). Anti-CD3/CD28 stimulation led to an overall increase in the secretion of IL-2, CXCL-10, granzyme B, and IFN-γ (Figure 6(B)). Simultaneously, stimulation decreased secretion of VEGF and IL-8 (associated with tumour progression). Variation in the response to stimulation within individual tumour tissues was detected for most cytokines, particularly CXCL10 (same symbol within one bar). Levels of PD-L1 (*p* = 0.03) and IL-17 (*p* = 0.06) secretion after stimulation were higher in the organ-adjacent margin than in the necrotic centre and free margin, respectively (Figure 6(C)). Moreover, increased IL-15 production (*p* = 0.1) was observed in the organ-adjacent margin. Consistent with the previous FAMD analysis, patient samples were well separated into clusters. However, no separate clusters were observed for tumour regions (Figure S3). T-cell stimulation led to the enhanced production of many cytokines, some of which were increased in the organ-adjacent margin; however, the variability in response to stimulation was not driven by the region where the sample was collected.

### In vitro efficacy of anti-LAG-3 therapeutics

**Figure 7.**
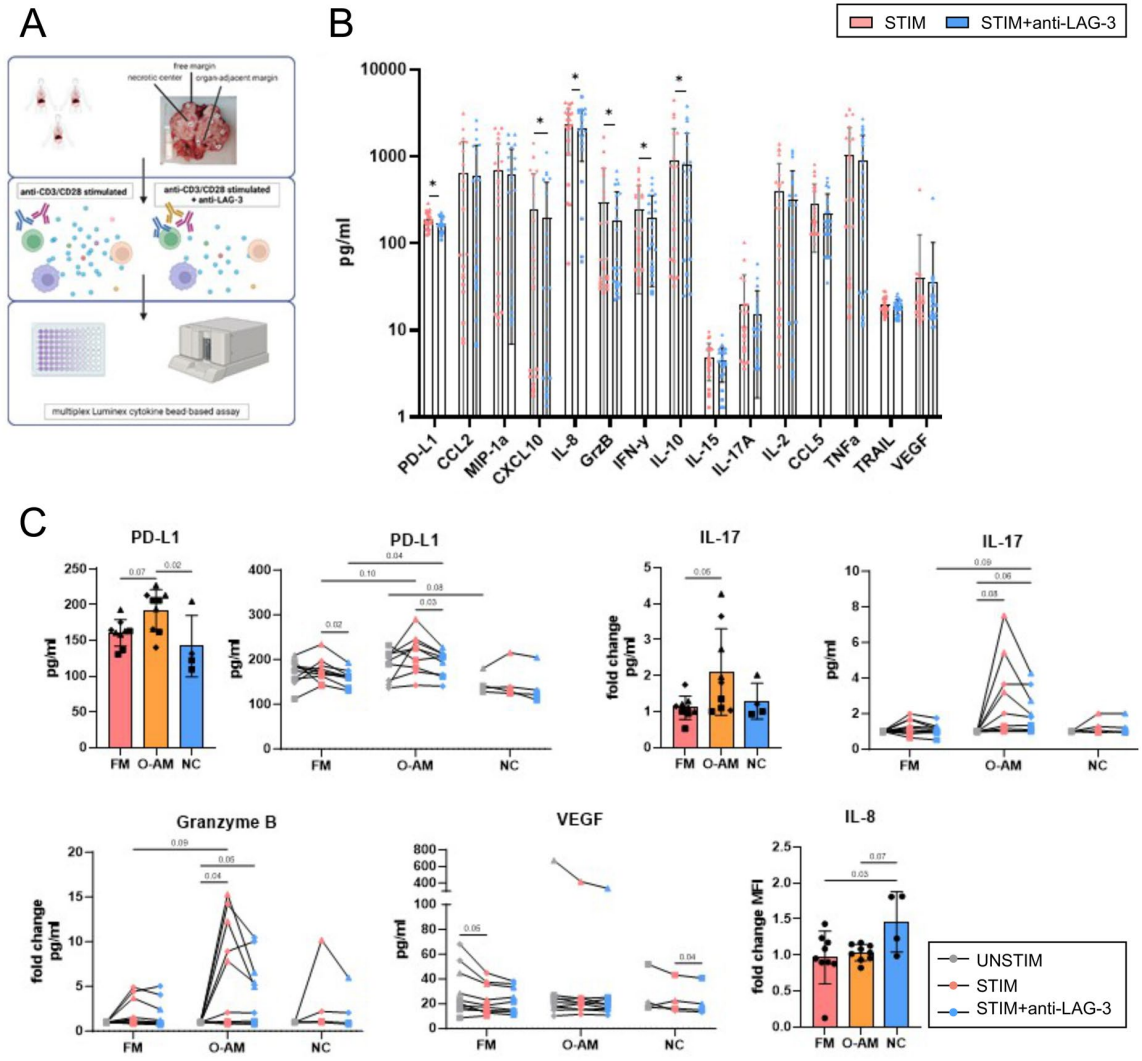
Analysis of intratumoral and region-specific immune response anti-LAG-3 blockade. (A) Experimental workflow. (B) Changes in cytokine secretion after anti-CD3/CD28 stimulation for 24 h with or without anti-LAG-3 blockade (*n* = 24). Significance was determined by multiple paired Wilcoxon tests and false discovery rate (FDR). FDR does not represent the actual *P*-value. (C) Region-specific cytokine secretion at the free margin (FM), organ-adjacent margin (O-AM), and necrotic centre (NC) under stimulated + anti-LAG-3 condition or under all three conditions (unstimulated, stimulated, and stimulated + anti-LAG-3). Within the stimulated + anti-LAG-3 group, the data were normally distributed; therefore, one-way ANOVA was performed. For the comparison between the tumour regions under various conditions, a two-way mixed-effects model with the Geisser-Greenhouse correction and Tukey’s multiple comparison test were utilized. *p* ≤ 0.05 was considered significant. The data are displayed as the mean with standard deviation. The samples from each patient are represented by a special symbol: patient B, square; patient C, triangle; patient D, rhombus. Image created using biorender.

As the tumour immune microenvironment can undergo substantial changes following therapeutic interventions, we evaluated the impact of anti-LAG3 therapy on the functional profiles of immune cells within the tumour tissue. Furthermore, we aimed to determine whether therapeutic responses are consistent across large tumours or whether region-specific differences in the immune response to anti-LAG3 blockade may exist. (Figure 7(A)). Anti-LAG-3 blockade led to lower cytokine production than that in stimulated samples (Figure 7(B)). Additionally, we observed substantial variation within each LMS tissue (same symbol within the bar). As shown in Figure 7(C), anti-LAG3 therapy resulted in increased PD-L1 secretion, which was particularly pronounced at the organ-adjacent margin compared with other regions; however, PD-L1 levels following anti-LAG-3 blockade were even higher in samples with simple T cell stimulation indicating that therapeutic interventions can substantially modulate the tumour microenvironment and, more importantly, influence the presence of PD-L1 within the TME. The fold change of IL-17 was higher at the organ-adjacent margin than at the free margin in both analyses (Figures 6(C), 7(C)). The enhanced reactivity of the organ-adjacent region was further evidenced by the production of granzyme B. Interestingly, the VEGF levels remained unchanged at this location under various conditions. IL-8 secretion was higher at the necrotic centre than at the free and organ-adjacent margins. Moreover, IL-2 and CXCL10 were positively correlated (Figure S4). The variability was driven by the patient rather than the tumour region; however, some distinct patterns of immunity were observed between the organ-adjacent margin and necrotic centre when all cytokines were compared.

## Discussion

The TME of LMS remains poorly understood, with insufficient data on its composition. The treatment of LMS with conventional therapies is limited, and patient outcomes remain poor despite these approaches. Thus, there is an urgent need to explore immunotherapy as a potential treatment option. However, the selection of optimal immunotherapeutic strategies requires a comprehensive understanding of the TME within LMS [34]. Only a limited number of studies have attempted to characterize the immune landscape within the TME of STS. Given the rarity and extreme histological heterogeneity of these tumours, most STS studies have combined various histological subtypes, resulting in limited data specifically dedicated to LMS. LMSs are usually classified as immune cold or even immune desert type of tumours [35,36]. Additionally, retroperitoneal LMS shows even lower infiltration of immune cells than LMS from extremities, uterine, and pelvic locations [37]. In contrast, 20%–38% of LMS tumours exhibited signatures of hot tumours based on the 90-gene signature or cell type identification by estimating relative subsets of RNA transcripts (CIBERSORT) method. Patients with such tumours may thus be suitable candidates for the treatment with ICIs [35].

In this study, we comprehensively analysed the phenotypes and subpopulations of T cells and macrophages. We identified a complex landscape of macrophage subsets within LMS involving both M1- and M2-like phenotypes. Interestingly, we did not observe a significant skew towards an M2-like immunosuppressive phenotype. Moreover, the number of M1-like macrophages was slightly higher in the organ adjacent margin. Small subpopulations of macrophages with an M1-like phenotype (CD163^low^CD206^low^PD-L1^low^SIRP-α^high^) were more abundant in the necrotic centre of the tumour, yet almost undetectable at the organ-adjacent margins; however, some M1-like macrophages also displayed PD-L1. Furthermore, elevated levels of IDO^+^ M2-like macrophages and increased PD-L1 secretion in the organ-adjacent margin were observed following stimulation, contrasting with the high proportion of M1 macrophages in this region. Additionally, significant levels of IL-15 and IL-17 were detected, indicating a potential conflict between the tumour’s immunosuppressive effects and the active antitumor immune response. We postulate that this dynamic may be influenced by increased tumour vascularization within the organ-adjacent margin [38]. Thus, our findings suggest that LMS has a complex and heterogeneous macrophage landscape and that LMS-associated macrophages retain plasticity, making them potential targets for therapeutic reprograming.

Our comprehensive spatial immunoprofiling of retroperitoneal LMS also revealed significant intratumoral heterogeneity in immune checkpoint molecule expression, which challenges the existing binary classification of these tumours as either immune-cold or immune-hot. On both T cells and non-T cells, we observed differential expression of PD-1, LAG-3, and TIM-3 molecules. PD-1, in particular, was highly expressed on both CD4^+^ and CD8^+^ T cells across all patients, with CD8^+^ T cells often co-expressing TIM-3. These findings are in accordance with various studies suggesting that therapies targeting PD-1, potentially along with TIM-3, could be beneficial [39]. Furthermore, the variability in CD8^+^ TIM-3^+^ T-cell frequencies (ranging from 5%–69%) within different areas of a single tumour suggests the potential for the coexistence of immune-active and immune-suppressed areas. CD8^+^ T cells expressing high levels of all three inhibitory molecules—PD-1, TIM-3, and LAG-3—were relatively sparse in most patients. Our analyses also revealed three distinct CD8^+^ T cell populations with higher LAG-3 expression. Notably, one of these populations, characterized by low TIM-3 and PD-1 expression, was found in higher proportions in 75% of patients, highlighting LAG-3 as a particularly compelling therapeutic target.

Of particular significance was the detection of substantial intratumoral variability in PD-1 and PD-L1 expression *via* immunohistochemistry given that these molecules are critical determinants of patient eligibility for immunotherapy of other malignancies in clinical practice. To which extent this immune heterogeneity could be present within smaller metastatic lesions, including pulmonary metastases, has not yet been systematically investigated in LMS. While this remains an open question, evidence from other tumour types suggests that even small lesions can harbour distinct immune profiles, raising the possibility that similar heterogeneity may also exist in LMS [40–42].

Patients in our cohort exhibited notable variability in PD-L1 expression across tumour regions, with patient A having high PD-L1 expression in the free margin, whereas other regions showed intermediate expression. Patients B, C, and D exhibited more heterogeneity, with patient B showing a wide range of PD-L1 expression across all regions and patient D showing PD-L1 expression only in the vital centre. Because PD-L1 expression can vary significantly within a single tumour, a sample obtained from a region with low or absent expression may not accurately reflect the overall PD-L1 status. This could lead to a patient being incorrectly classified as ineligible or predicted to respond poorly to immunotherapy, even if other regions of the tumour exhibit higher levels of PD-L1 expression. Therefore, assessing the PD-L1 status in multiple regions of the tumour is essential to avoid misclassification and to ensure appropriate treatment decisions. This observation could also help explain why some patients with cancer in clinical trials responded to immunotherapy with pembrolizumab, despite extremely low or undetectable levels of PD-L1 expression [24].

This agrees with the findings of Kwong et al. who described heterogeneous PD-L1 expression in different tumour regions within lung adenocarcinomas [43]. Therefore, PD-L1 status from a single biopsy may not be a useful biomarker for immunotherapy in large LMSs.

The PEMBROSARC study evaluating the efficacy of pembrolizumab combined with low-dose cyclophosphamide in patients with advanced STS demonstrated that patients with TLS-positive STS achieved an objective response rate of 30%, which is comparable to the response rates seen in approved indications, such as lung cancer and melanoma [44]. However, in approximately 80% of patients, TLS is absent in sarcomas, which presents a significant challenge in expanding these immunotherapeutic approaches for sarcoma patients [45]. Therefore, we examined TLS across diverse regions of tumour tissue. Although the number of LMS cases in this cohort was limited, TLSs were detected exclusively at the tumour margins and in a limited number of samples.

Our findings, together with our previous results, underscore the challenge of detecting TLS in large LMS, as a single biopsy may not fully capture the entire spectrum of immune cell infiltration and TLS [46]. This limitation raises concerns about the potential for incomplete evaluations and opens discussions on whether patients initially classified as TLS-negative might still harbour TLS in specific regions. Additionally, this study raises the question of whether the presence of TLS in these localized areas alone could predict a favourable response to immunotherapy or whether the presence of TLS across multiple tumour regions is necessary for a patient to respond effectively.

Previously, high LAG-3 expression was detected in STS patients with TLS [36]. However, elevated LAG-3 density is correlated with poor survival in STS patients [47]. LAG-3 inhibitors are currently being evaluated in combination with anti-PD-1 in advanced STS patients [45]. We observed differences in LAG-3 expression between the free and organ-adjacent margins in T cells and non-T cells. Although LAG-3 is mostly associated with T cells, its presence has been described in activated natural killer (NK) cells, B cells, and plasmacytoid dendritic cells [48]. Lower expression of LAG-3 in the organ-adjacent margin led to enhanced secretion of PD-L1 and IL-17 after anti-LAG-3 blockade compared with other regions and free margins, respectively. These results imply that different levels of LAG-3 expression and overall different compositions of specific tumour regions may lead to diverse responses to immune checkpoint blockade. This is further supported by large differences in cytokine production after anti-LAG-3 blockade in the same tumour tissue.

FAMD revealed that tumour region-specific patterns existed within large LMSs; however, interpatient variability was higher. Swisher et al. described a similar phenomenon in lung cancer. In this study, interpatient variability was higher than intratumoral heterogeneity. The main intratumoral differences in lung cancer were noted in immune cells and stromal cells. For example, within a single tumour tissue, both enrichment and decline in PD-1 resistance co-enrichment scores were detected [49]. In a transcriptome analysis of LMS tumours from 22 patients, Feng et al. identified intratumoral heterogeneity in only 4 cases; however, the immune signature (hot or cold) was homogeneous across the tumour tissue [35]. Until now, Lee et al. represented the most extensive evaluation of intratumoral heterogeneity in LMS, analysing over 40 samples *via* Tissue Microarrays. In this study, CD3, CD4, CD8, and CD20 IHC-positive lymphocytes were evaluated from formalin-fixed, paraffin-embedded tissue across five regions per tumour. In contrast, we used fresh tumour tissues and investigated 10 distinct regions with a broader methodology—including cell culture, FACS, multiplex Luminex assays, and IHC—to assess TIL subpopulations, macrophages, and TLS. In addition, this study is the first to evaluate the effect of anti-LAG-3 therapy on LMS *in vitro* and demonstrates that therapeutic interventions can exert a profound impact on the tumour microenvironment and, in particular, shape the abundance of PD-L1 within the TME. Similar to our findings, Lee et al. reported greater interpatient variability than intratumoral heterogeneity in LMS, with 79% of cases showing homogeneity in TILs across sampled regions. Additionally, they noted marked differences in T-cell densities between tumour margins and cores in 4 out of 6 patients, emphasizing spatial immune variation within tumours [50].

Our study has several limitations. One limitation of this study is the inconsistency of patient samples; some were either unsuitable for analysis or did not provide enough cells for all experiments. Another limitation of this study is the relatively small cohort size. Although 40 tissue samples were analysed, these were obtained from only four patients, which inevitably limits the generalisability of our findings. Nonetheless, given the rarity of LMS and the scarcity of available tumour material, this study provides a valuable proof-of-concept demonstrating the feasibility and importance of spatially resolved immune profiling. Future studies involving larger, multi-institutional cohorts will be critical to validate and expand upon these initial observations. Further research is needed to elucidate the mechanisms driving the observed intratumoral heterogeneity, particularly the roles of genetic mutations, epigenetic changes, and environmental factors within the TME.

Our study did not extensively explore the contributions of other immune cells, such as B cells, natural killer cells, and dendritic cells, which may offer a more comprehensive understanding of tumour-immune interactions in LMS. We did not analyse all immune checkpoint molecules, including CTLA-4, because its expression levels have not consistently demonstrated a correlation with therapeutic outcomes in clinical studies [51]. Investigating these factors in future studies could help identify mechanisms of immune evasion and resistance, ultimately informing the development of more effective therapeutic strategies that enhance the efficacy of immunotherapy in LMS. It is also important to acknowledge that our findings are based on analyses performed at a single time point. This cross-sectional design does not capture the temporal dynamics of the tumour microenvironment, including potential fluctuations in cellular populations that may occur naturally over time or in response to therapeutic interventions. Also, our current dataset did not allow for a direct assessment of both primary and metastatic lesions in LMS, as we could only analyse primary tumours and not metastases. Given that previous studies have demonstrated substantial discordance in PD-L1, PD-1, PD-L2, and TIL levels between primary tumours and their paired metastases, it is reasonable to postulate that metastatic lesions within the same LMS patient may likewise harbour distinct immune populations, potentially contributing to mixed clinical responses to immunotherapy [52]. Such intratumoral heterogeneity has been documented between primary tumours and metastatic lesions in cancers including non-small cell lung cancer, colorectal cancer, and renal cell carcinoma but has not yet been reported in LMS [53–55].

While our review of available clinical trial data on immunotherapy in LMS did not reveal systematic reporting of mixed responses, overall responses to immunotherapy in this disease have been notably poor and, in some instances, heterogeneous. For example, in the SARC028 trial, no patients with leiomyosarcoma achieved an objective response; however, 60% of patients treated with pembrolizumab experienced stable disease, whereas 40% exhibited progressive disease. As highlighted by Tawbi et al. the mechanisms underlying resistance to immunotherapy in LMS remain poorly understood. Nevertheless, we believe that our data provide important insights into this issue by revealing immune heterogeneity that may help explain such variable outcomes [56]. Our findings emphasize the importance of collecting multiple biopsy samples to precisely evaluate the immune landscape. This is particularly crucial for large tumours, where immune heterogeneity can vary significantly across regions of the tumour. Relying on a single biopsy may not capture the full spectrum of immune cell infiltration and activity, potentially leading to an incomplete or inaccurate assessment of the TME. Therefore, we propose collecting four to six biopsies from each tumour including samples from both the tumour centre and its margins whenever feasible. Moreover, the use of advanced immunoprofiling techniques, including multiplexed assays and spatial imaging, can provide insights into the complex interplay of immune cells within the TME. However, the high cost and technical complexity of these approaches currently limit their routine clinical implementation. Future work should focus on improving feasibility and cost-effectiveness, to facilitate the translation of these techniques into personalized therapeutic strategies for patients.

## Conclusion

Our study highlights the importance of recognizing and addressing intratumoral heterogeneity in retroperitoneal LMS. The variability in immune cell infiltration and checkpoint molecule expression across different tumour regions presents challenges and opportunities for optimizing immunotherapy. A more nuanced understanding of the TME, coupled with the development of region-specific and combination treatment approaches, can improve outcomes for patients with LMS.

## Supplementary Material

supplementarymaterialToSubmit FIN.docx

Sup Fig 1.JPG

Sup Fig 2.JPG

Sup Fig 3.JPG

Sup Fig 4.JPG

## Data Availability

Data are available from the corresponding author upon reasonable request.
