# Supplementary material for: Spatial immunoprofiling of retroperitoneal leiomyosarcomas reveals intratumoral heterogeneity in immune cell infiltration, checkpoint molecule expression, and tertiary lymphoid structures
Source: Ann Med. 2025 Oct 13;57(1):2568725. doi: 10.1080/07853890.2025.2568725 (PMC12519586; doi:10.1080/07853890.2025.2568725)
Supplement: supplementarymaterialToSubmit FIN.docx [file IANN_A_2568725_SM5638.docx]

**Supplementary Tables**

Table S1. Patient characteristics

| **Patient** | **A** | **B** | **C** | **D** |
| --- | --- | --- | --- | --- |
| Sex | Female | Female | Male | Female |
| Age (y) | 76 | 55 | 70 | 73 |
| Tumor stage | T3 | T4 | T3 | T4 |
| Tumor grade | G3 | G3 | G3 | G2 |
| Tumor size (mm) | 126 × 94 × 65 | 280 × 232 × 159 | 129 × 81 × 64 | 180 × 120 × 115 |
| Status at last follow up | Alive  (37 months) | Alive  (31 months) | Alive  (28 months) | Alive  (27 months) |
| Disease recurrence | Yes | No | No | No |
| Time to recurrence | 26 months | N/A | N/A | N/A |
| Metastases | Yes (lungs) | No | No | No |

Table S2. Tumor grade for each tumor sample

| **Location ↓ Patient →** | **A** | **B** | **C** | **D** |
| --- | --- | --- | --- | --- |
| Free margin | G2 | G2–3 | G2–3 | G1–2 |
|  | G2 | G3 | G3 | G2 |
|  |  | G3 | G3 |  |
|  |  | G3 | G2–3 |  |
| Organ-adjacent margin | G2 | G3 | G3 | G2 |
|  | G3 | G3 | G2–3 | G2 |
|  | G3 | G2–3 | G3 | G2 |
|  |  |  |  |  |
| Vital center | G2–3 | G2 | G2–3 | G2 |
|  |  |  |  | G2 |
|  |  |  |  | G1–2 |
|  |  |  |  | G2 |
| Necrotic center | G2–3 | G3 | G2–3 | G2 |
|  |  |  | G3 |  |

| **Target** | **Conjugate** | **Clone** | **Dilution** | **Manufacturer** |
| --- | --- | --- | --- | --- |
| **T cells – extracellular staining** | | | | |
| CD3 | PE-Dylight 594 | MEM57 | 1:25 | Exbio |
| CD4 | PerCP | MEM241 | 1:25 | Exbio |
| CD8 | FITC | MEM31 | 1:25 | Exbio |
| FasL | PE | NOK-1 | 1:17 | Biolegend |
| LAG-3 | PE-Cy7 | 11C3C67 | 1:17 | Biolegend |
| PD-1 | BV421 | EH12.2H7 | 1:17 | Biolegend |
| TIM-3 | APC | A18087E | 1:17 | Biolegend |
| **Macrophages – extracellular staining** | | | | |
| CD45 | BV510 | HI30 | 1:25 | Biolegend |
| CD86 | BV605 | BU63 | 1:17 | Biolegend |
| CD163 | PE-Cy7 | GHI/63 | 1:17 | Biolegend |
| CD206 | PerCP-Cy5.5 | 15-2 | 1:25 | Biolegend |
| SIRP-α | PE | 15-414 | 1:17 | Biolegend |
| PD-L1 | BV650 | 293.2A3 | 1:17 | Biolegend |
| VISTA | BD Horizon BV421 | MIH65 | 1:17 | BD Biosciences |
| **Macrophages – intracellular staining** | | | | |
| CD68 | FITC | YI/82A | 1:25 | Biolegend |
| IDO | Alexa 647 | V50-18186 | 1:17 | BD Biosciences |

Table S3. List of antibodies for flow cytometry

Table S4. Minimal and maximal density of PD-1 and PD-L1 in each patient

| **Patient** | **PD-1 min-max (cells/mm^2^)** | **PD-L1 min-max (cells/mm^2^)** |
| --- | --- | --- |
| A | 9 – 1674 | 27 - 504 |
| B | 252 - 1479 | 2 - 12 |
| C | 16 - 198 | 0 - 32 |
| D | 6 - 155 | 0 - 8 |

Table S5. Median percentage of region-specific immune cell populations

|  | **CD4^+^ out of CD3^+^** | **CD8^+^ out of CD3^+^** | **PD-1^+^ out of CD3^+^** | **LAG-3^+^ out of CD3^+^** | **TIM-3^+^ out of CD3^+^** | **PD-1^+^ out of CD3^-^** | **LAG-3^+^ out of CD3^-^** | **TIM-3^+^ out of CD3^-^** | **M1-like macrophages out of CD68^+^** | **M2-like macrophages out of CD68^+^** |
| --- | --- | --- | --- | --- | --- | --- | --- | --- | --- | --- |
| Free margin | 43% | 31% | 70.5% | 18.3% | 10.4% | 16.4% | 25.9% | 14.7% | 50.3% | 49.4% |
| Organ-adjacent margin | 47% | 36.9% | 67.8% | 9.8% | 15.6% | 7.5% | 14.9% | 14% | 56.2% | 43.6% |
| Vital center | 40% | 46.8% | 68.1% | 11.1% | 27.2% | 4% | 5.3% | 5.9% |  |  |
| Necrotic center | 51.3% | 22.7% | 63.4% | 21.2% | 8.7% | 10.2% | 21.7% | 11.2% | 44% | 55.9% |

**Supplementary Figures**

**Supplementary Figure 1** Experimental workflow: Four retroperitoneal treatment-naive leiomyosarcomas were surgically resected and a sarcoma-experienced pathologist evaluated the tumor tissue and collected ten samples from each tumor within four defined regions (vital and necrotic centers and free and organ-adjacent margins). The tumor tissues were formalin-fixed paraffin-embedded (FFPE) or enzymatically and mechanically dissociated for further immune profiling. Flow cytometry was performed with two panels that investigated T cell and macrophage subsets. FFPE tissues were stained for PD-1 and PD-L1 and inspected for the presence of tertiary lymphoid structures. Unstimulated, anti-CD3/CD28 stimulated, and anti-CD3/CD28 stimulated+anti-LAG-3 samples were analyzed by a multiplex Luminex cytokine bead-based assay. Image created in Biorender.

**Supplementary Figure 2** Percentage of PD-L1 positive tumor cells in analyzed samples. Four patients (A–D) and four tumor regions (free margin, organ-adjacent margin, vital center, and necrotic center) were examined for the percentage of PD-L1 positive tumor cells (*n* = 35). Complete or partial membrane positivity, regardless of intensity was considered positive. Based on the percentage of positive tumor cells, each sample was semi-quantitatively classified into three categories; no expression (< 1% of positive cells, red), intermediate expression (1% to 49% of positive cells, light green), and high expression (> 50% of positive cells, dark green).

**Supplementary Figure 3** Factorial analysis of mixed data (FAMD) was performed for categorical (patient and tumor region) and continuous variables (cytokines, pg/mL). **(A)** Correlative circle marks quantitative variables; each arrow depicts the contribution of the variable to the first and second dimensions. Moreover, the circle illustrates the correlations between the variables. **(B)** Individual points are plotted and colored based on patient (B, yellow; C, red; D, blue) and location (free margin, red; necrotic center, blue organ-adjacent margin, orange). The largest point shows the average location of each group.

**Supplementary Figure 4** Factorial analysis of mixed data (FAMD) was used to evaluate categorical (patient and tumor region) and continuous variables (cytokines, pg/mL). **(A)** Correlative circle marks quantitative variables; each arrow depicts the contribution of the variable to the first and second dimensions. Moreover, the circle illustrates the correlations between the variables. **(B)** Individual points are plotted and colored based on patient (B, yellow; C, red; D, blue) and location (free margin, red; necrotic center, blue; organ-adjacent margin, orange). The largest point shows the average location of each group.
