# Supplementary figures and images for: Spatial immunoprofiling of retroperitoneal leiomyosarcomas reveals intratumoral heterogeneity in immune cell infiltration, checkpoint molecule expression, and tertiary lymphoid structures
Source: Ann Med. 2025 Oct 13;57(1):2568725. doi: 10.1080/07853890.2025.2568725 (PMC12519586; doi:10.1080/07853890.2025.2568725)

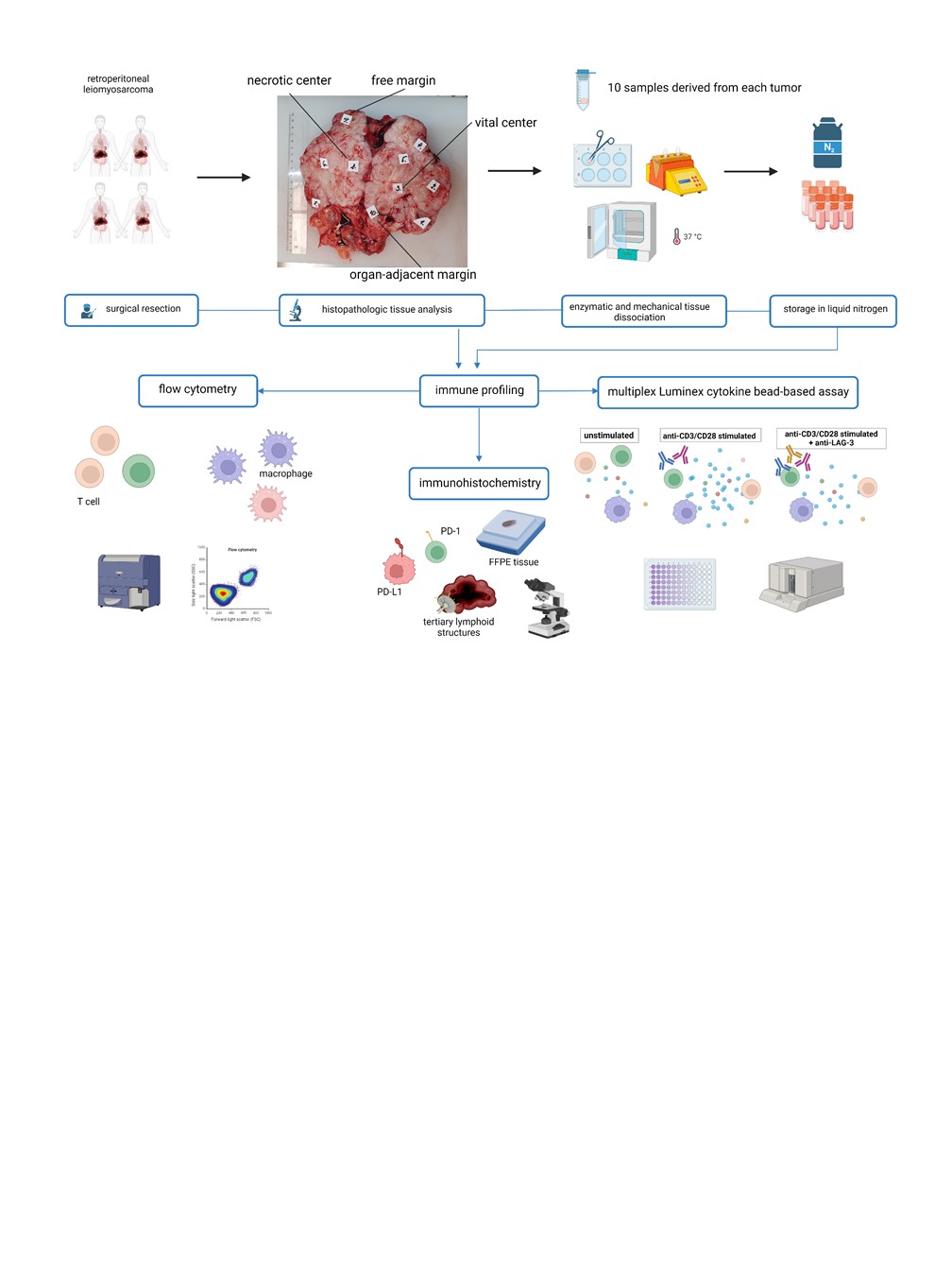

Supplement: Sup Fig 1.JPG [file IANN_A_2568725_SM5637.jpg]

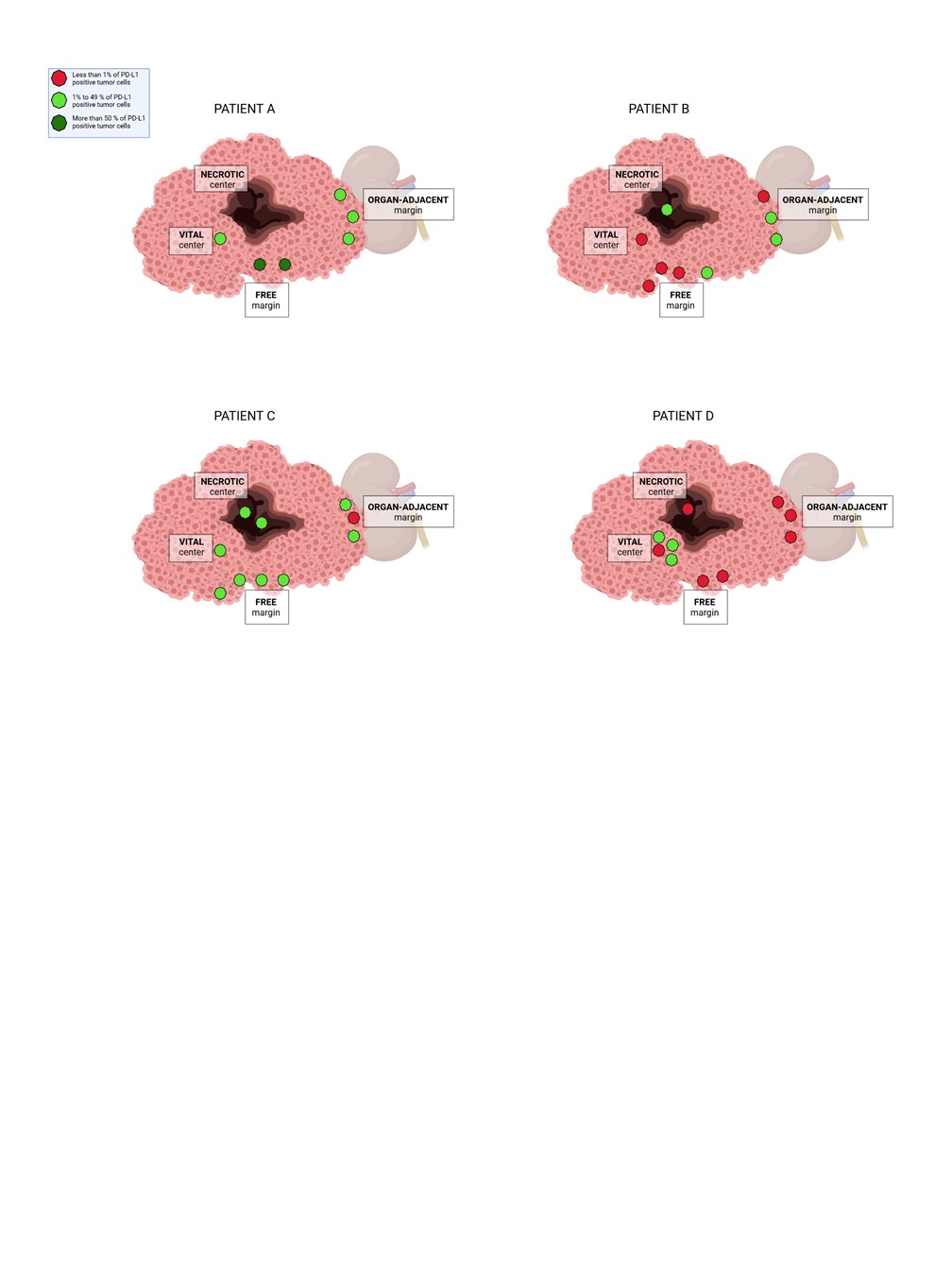

Supplement: Sup Fig 2.JPG [file IANN_A_2568725_SM5636.jpg]

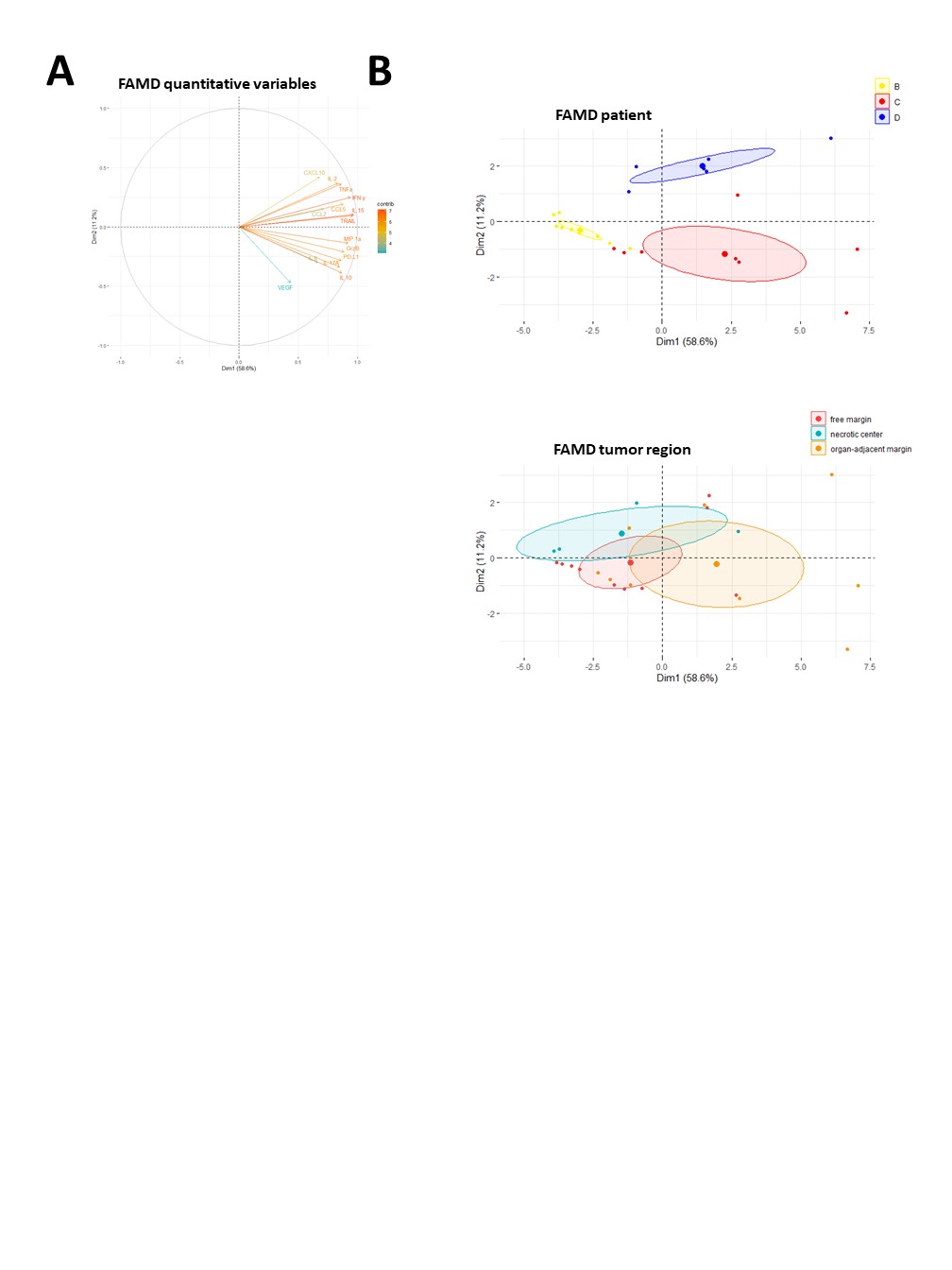

Supplement: Sup Fig 3.JPG [file IANN_A_2568725_SM5635.jpg]

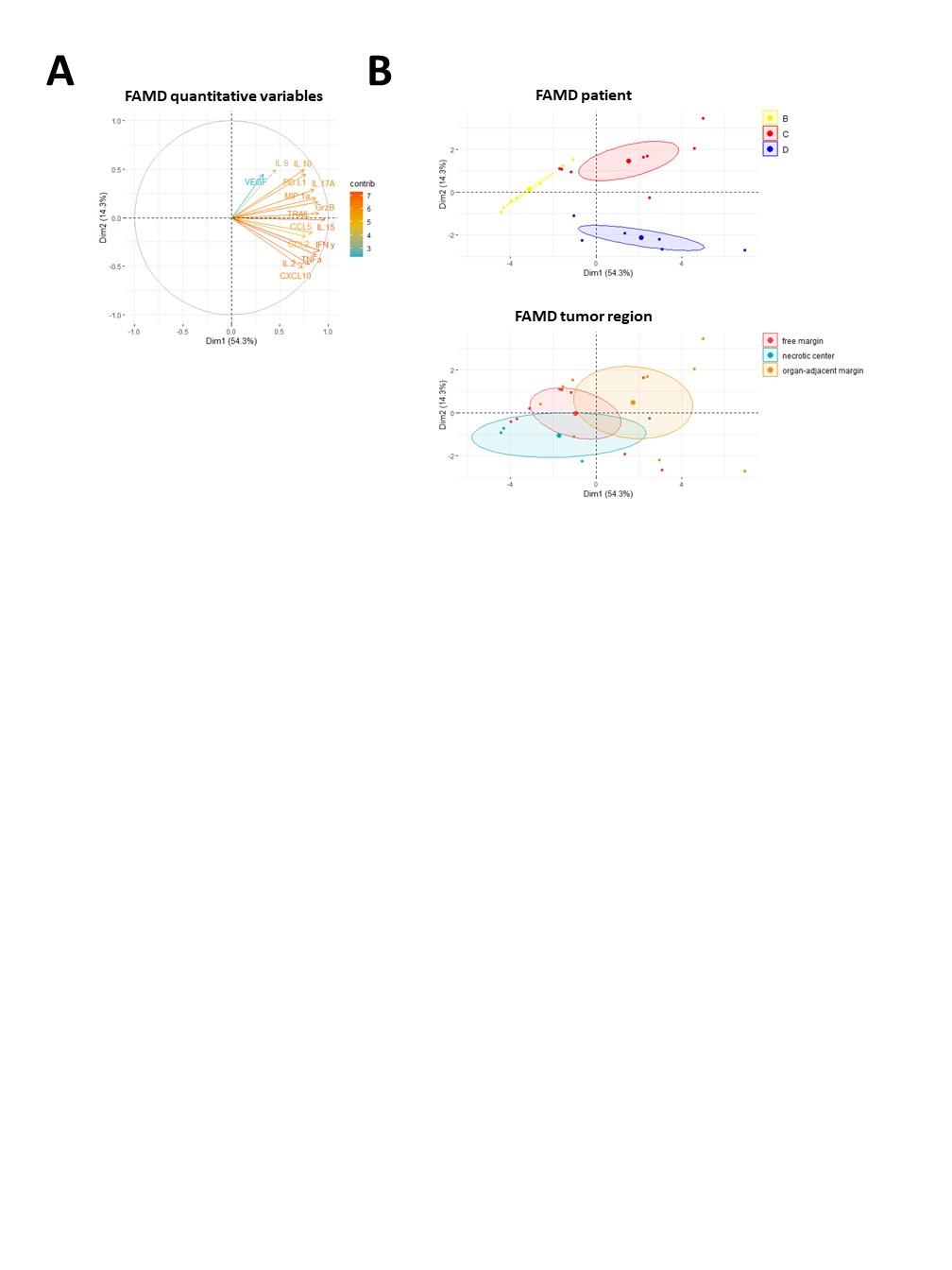

Supplement: Sup Fig 4.JPG [file IANN_A_2568725_SM5634.jpg]
